# Unexpected Cardiac Arrest During MRI in a Patient With Acute Brainstem Infarction: A Case Report and Imaging Insights

**DOI:** 10.7759/cureus.81691

**Published:** 2025-04-04

**Authors:** Hiroyuki Tokue, Azusa Tokue, Masashi Ebara, Yoshito Tsushima

**Affiliations:** 1 Department of Diagnostic Radiology and Nuclear Medicine, Graduate School of Medicine, Gunma University, Maebashi, JPN

**Keywords:** acute brainstem infarction, cardiac arrest, mra, mri, t2-weighted imaging

## Abstract

We report the case of a 65-year-old man with a history of hypertension and diabetes who experienced cardiac arrest during MRI for acute brainstem infarction. Initial imaging revealed right vertebral artery occlusion and brainstem infarction. During follow-up MRI because of worsening symptoms, magnetic resonance angiography revealed an absence of cerebral arterial flow, and T2*-weighted imaging revealed arterial and venous engorgement, which were later recognized as early signs of cardiac arrest. Cardiopulmonary resuscitation was initiated, but hypoxic encephalopathy persisted. This case highlights the importance of continuous vital sign monitoring and real-time image evaluation during MRI, particularly in high-risk patients with stroke.

## Introduction

Acute ischemic stroke, particularly brainstem infarction, poses a significant risk of rapid neurological deterioration and life-threatening complications [1]. Given the brainstem’s critical role in autonomic functions, even a minor ischemic event can disrupt cardiovascular and respiratory control [2]. MRI is essential for diagnosing and monitoring stroke progression, yet it presents unique challenges, including prolonged scan times and the necessity of maintaining patient immobility. These factors can obscure early physiological deterioration, making real-time monitoring indispensable.

In this report, we present a rare case of cardiac arrest occurring during MRI in a patient with acute brainstem infarction. Although resuscitation was successful, the patient developed hypoxic encephalopathy. Notably, retrospective image analysis revealed key findings on magnetic resonance angiography (MRA) and T2*-weighted imaging (T2*-WI) that were initially overlooked, clues that could serve as early warning signs of circulatory collapse. This case underscores the critical need for continuous vital sign monitoring and real-time image assessment during MRI, especially in high-risk stroke patients, to facilitate timely intervention and improve patient outcomes.

## Case presentation

The patient was a 65-year-old man with a history of hypertension, diabetes mellitus, and smoking (smoking 20 cigarettes per day for over 30 years). He also had a history of transient ischemic attack, placing him at high risk for stroke. Two days before hospital admission, he had been experiencing mild headaches but continued daily activities without concern.

On the morning of admission, he suddenly experienced dizziness and nausea, restricting his ability to move independently. His family called for an ambulance, and he was transported to the hospital. On arrival, his vital signs were as follows: blood pressure, 170/95 mmHg; pulse, 90 beats/minute; respiratory rate, 18 breaths/minute; and oxygen saturation, 97%. Neurologically, left-sided motor paralysis, dysphagia, and dysarthria were observed, raising the suspicion of acute ischemic stroke. His level of consciousness was normal, and no visual field defects, sensory deficits, or aphasia were noted. The National Institutes of Health Stroke Scale score was estimated to be 6 points. Given that the time of symptom onset was unclear and exceeded the standard thrombolysis window, intravenous tissue plasminogen activator was not administered.

**Figure 1:**
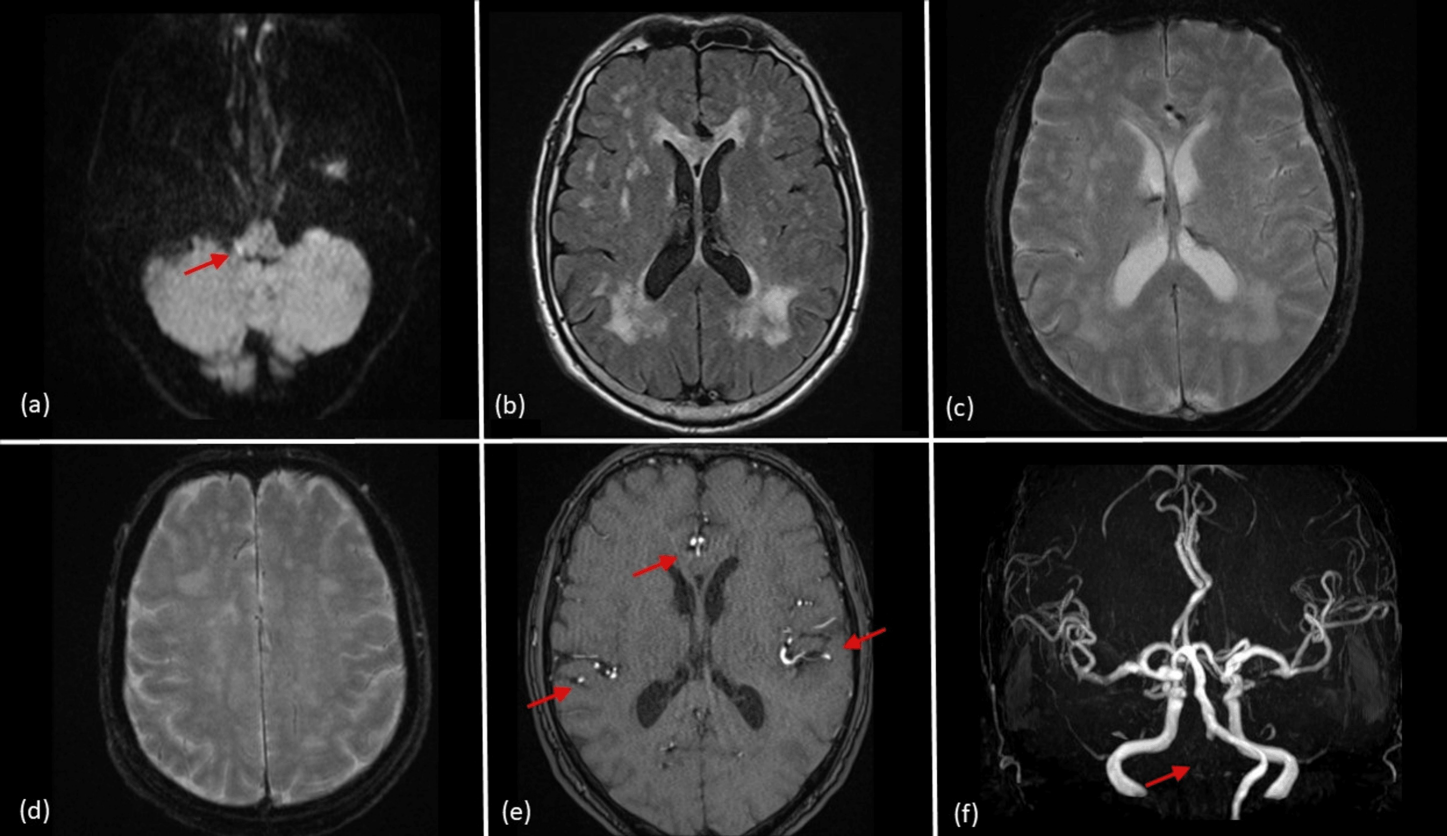
Initial MRI findings on hospital admission. (a) Diffusion-weighted imaging demonstrates acute ischemic changes in the right brainstem, with restricted diffusion in the right vertebral artery territory (arrow). (b) Fluid-attenuated inversion recovery image shows chronic ischemic changes in cerebral white matter. (c) T2-weighted imaging (T2*-WI) depicts no abnormal venous or arterial engorgement at this stage. (d) Another slice of T2*-WI shows no significant vascular abnormalities. (e) The source images of magnetic resonance angiography (MRA) show normal arterial blood flow signals except for the right vertebral artery (arrows). (f) Maximum intensity projection of the MRA highlights the absence of flow in the right vertebral artery, correlating with brainstem ischemia (arrow).

An urgent MRI revealed occlusion of the right vertebral artery and acute ischemic changes in the brainstem, although no significant abnormalities in cerebral blood flow were observed (Figures 1a-1f). The MRI findings were initially interpreted by a radiologist. Given the clinical suspicion of acute ischemic stroke, a neurologist also reviewed the imaging and was involved in the patient’s management.

**Figure 2:**
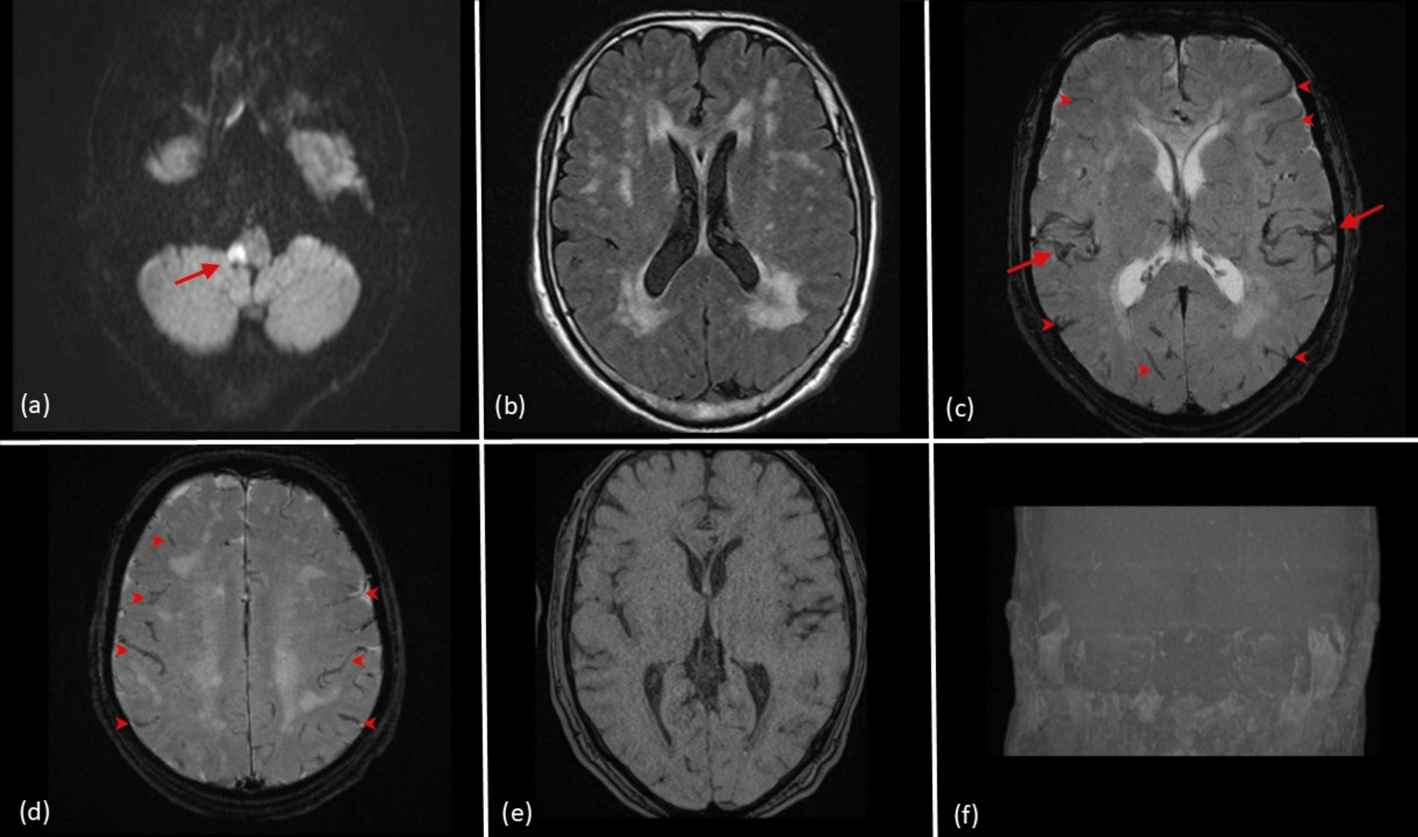
MRI findings during a follow-up scan. (a) Diffusion-weighted imaging shows a slight expansion of the ischemic area, with increased diffusion restriction in the right brainstem compared to the initial scan (arrow). (b) Fluid-attenuated inversion recovery image remains largely unchanged from the initial scan. (c) T2*-weighted imaging (T2*-WI) shows dilated cerebral arteries (arrows) and veins (arrow heads) as low signal intensity. (d) Another T2*-WI slice shows venous congestion as low signal intensity (arrow heads). (e) Magnetic resonance angiography (MRA) source image displays the absence of arterial blood flow signals, which was initially misinterpreted as a technical malfunction. (f)  Maximum intensity projection of the MRA confirms the complete loss of cerebral arterial flow, correlating with the patient’s cardiac arrest.

However, his symptoms worsened in the afternoon, prompting a follow-up MRI. In the afternoon MRI (six hours after the first MRI procedure), the patient was still able to move to the examination room with assistance, although he required support for walking. The MRI protocol included diffusion-weighted imaging (DWI), fluid-attenuated inversion recovery (FLAIR), T2-WI, T1-WI, MRA, and T2*-WI sequences. DWI revealed a slight expansion of the ischemic area (Figure 2a), and FLAIR showed no significant differences from the first scan (Figure 2b). During MRA, the technician noticed an absence of arterial blood flow signals but assumed it was a technical malfunction and continued the imaging. T2*-WI showed dilation of the cerebral arteries and veins, which went unnoticed at the time, but in retrospect, may have indicated venous congestion due to cardiac arrest (Figures 2c, 2d). Upon repeating MRA, arterial blood flow signals were still absent (Figures 2e, 2f).

At this point, the technician observed the abnormality and checked the patient, who was found to be in cardiac arrest. Cardiac arrest was estimated to have occurred within approximately 10 minutes of scanning. Immediate cardiopulmonary resuscitation was initiated, and spontaneous circulation was restored after approximately 10 minutes. However, the patient aspirated and subsequently developed hypoxic encephalopathy. Intensive care was continued, and the patient was transferred to another facility one month later.

## Discussion

This is an extremely rare case of cardiac arrest that occurred during MRI scanning in a patient with acute ischemic stroke. Notably, important findings related to blood flow and vascular dilation associated with cardiac arrest were overlooked on MRA and T2*-WI, which delayed early intervention. To prevent such incidents, it is crucial to continuously monitor the vital signs of patients during MRI and to evaluate the images in real time during the scan.

Brainstem infarction poses a high risk of rapid deterioration because of its potential to directly affect circulatory and respiratory functions. In this case, the condition of the patient worsened, necessitating a second MRI scan; however, vital signs were not monitored during the scan. For high-risk patients, continuous electrocardiogram and oxygen saturation monitoring should be implemented during MRI scans [3]. In addition, immediate evaluation of MRA and T2*-WI findings during the scan may facilitate earlier recognition of critical circulatory changes.

Furthermore, it is imperative to carefully assess MRI images as they are acquired while paying close attention to abnormal findings. In this case, the disappearance of arterial blood flow signals on MRA was observed but mistakenly attributed to a technical issue, delaying the recognition of cardiac arrest. As MRI can be time-consuming, especially in patients requiring prolonged scanning, real-time evaluation of images and interruption of scans when abnormalities are detected can improve patient outcomes.

Cardiac arrest during MRI in patients with acute ischemic stroke is exceptionally rare, with no similar cases reported in the existing literature. However, previous reports have documented hypointense signals in the cerebral arteries and veins on susceptibility-weighted imaging during arterial or venous congestion [4]. An intravascular thrombus manifests as an abnormal dark signal, referred to as the susceptibility vessel sign. This characteristic finding is marked by a thickened vessel with a dark signal, indicative of deoxyhemoglobin accumulation within trapped red blood cells inside the occluded vessel [5].

In the present case, the increased visibility of the veins on T2*-WI was likely due to a combination of factors. Stagnation of the cerebral arteries and elevated cerebral venous pressure, presumably resulting from cardiopulmonary arrest, likely contributed to hypoperfusion of the ischemic tissue. This may have led to an increase in oxygen extraction, resulting in elevated deoxyhemoglobin levels and enhanced susceptibility effects [4].

Findings such as the cessation of cerebral arterial blood flow on MRA and abnormal artery/venous congestion on T2*-WI, as seen in this case, may be useful for the early detection of cardiac arrest. This case underscores the importance of real-time monitoring during MRI, especially in patients at risk of sudden deterioration, such as those with a brainstem infarction, which affects circulation and respiration. Additionally, consistent real-time evaluation of MRI images enables the early detection of unexpected abnormalities, facilitating timely intervention, and improving patient prognosis.

Furthermore, this case suggests that MRA and T2*-WI may be valuable tools for the early detection of cardiac arrest. Comprehensive management, combining imaging diagnostics and monitoring, is necessary for similar cases.

## Conclusions

This rare case of cardiac arrest during MRI scanning in a patient with acute ischemic stroke emphasises the critical importance of monitoring vital signs and evaluating images in real time during the scan. Moreover, abnormal MRA and T2*-WI findings during cardiac arrest suggest the need for prompt evaluation of these images and immediate response to improve patient outcomes.

## Appendices

Technical information about the MRI

Brain MRIs were performed at 3T (Siemens Magnetom Prisma, Erlangen, Germany) using a 64-channel receive head/neck coil. The settings used were as follows: (a) DWI (repetition time (TR), 3,000 ms; echo time (TE), 65.3 ms; maps of the apparent diffusion coefficient (ADC) were generated using the b-values of 0 s/mm2 and 1000 s/mm^2^); (b) FLAIR (TR, 8,800 ms; TE, 94 ms; inversion time, 2,500 ms; slice thickness, 5 mm; intersection gap, 1 mm; field of view (FOV), 24 × 24 cm^2^; matrix number, 512 × 512); (c) T1-WI (TR, 550 ms; TE, 15 ms; flip angle, 70°; slice thickness, 5 mm; intersection gap, 0 mm; FOV, 24 × 24 cm^2^; matrix number, 512 × 512); (d) T2-WI (TR, 4500 ms; TE, 105 ms; flip angle, 90°; slice thickness, 5 mm; intersection gap, 0 mm; FOV, 24 × 24 cm^2^; matrix number, 512 × 512); (e) T2*-WI (TR, 27 ms; TE, 20 ms; flip angle, 10°; slice thickness, 5 mm; intersection gap, 0 mm; FOV, 24 × 24 cm^2^; matrix number, 512 × 512); (f) time of flight MRA (TR, 21 ms; TE, 2.3 ms; flip angle, 20°; slice thickness, 0.6 mm; intersection gap, 0 mm; FOV, 24 × 24 cm^2^; matrix number, 256 × 256).
